# P17 All in the Marrow - A case of Primary Bone Marrow Oedema

**DOI:** 10.1093/rap/rkac067.017

**Published:** 2022-09-28

**Authors:** Varghese Koshy, Amit Sharma, Vandana Gangadharan, Dharmesh Paliwal, George Koshy

**Affiliations:** Department of Rheumatology & Clinical Immunology, Command Hospital Southern Command, Pune, India; Department of Nuclear Medicine, Command Hospital Southern Command, Pune, India; Department of Pathology, NRI Institute of Medical Sciences, Visakhapatnam, India; Department of Nuclear Medicine, Command Hospital Southern Command, Pune, India; Department of Community Medicine, INHS Kalyani, Visakhapatnam, India

## Abstract

**Introduction/Background:**

Primary bone marrow oedema syndrome is an elusive and less well-defined entity. Whether Rheumatologists should consider it as a stand alone diagnosis, is debatable. It possibly would be best described as an MRI feature which could be a finding in a number of diseases which would include the initial phases of Osteonecrosis of the bone, Rheumatoid Arthritis, Spondyloarthritis, Enthesitis related, Post traumatic, OA, Infections and Cancers. The treatment options become constricted due to the paucity of evidence.

Rheumatologists need to consider this as an area of unmet need with development of consensus classification criteria and treatment approaches.

**Description/Method:**

27-year-old male, Height 174 cms Weight 90 Kgs BMI 29 Kg/m2, became symptomatic in Jan 2022 with complains of pain in the both hip joints & groin regions, pain became excruciating and he became bed-bound, with early morning stiffness lasting approximately 45 mins.

Had received steroids for COVID infection in August 2020.

Investigations

Hb 13.5gm/dl TLC 7000/mm^3^ Platelet 400 × 10^3^/mm^3^

Sr Bil 0.8mg/dl AST 16 IU/L. ALT 24 IU/L Sr Creatininine 1.1mg/dl

Blood Sugar Levels, Fasting 89 mg/dl Post Prandial 102 mg/dl

ESR 10 mm in 1st hour by Wintrobes method CRP Quantitative 29.38mg/L

HLA B27 by PCR Negative, RF Negative, ACCP Negative

Serum, IgG, Beta2 Glycoprotein 1.44 SGU Serum, IgM, Beta2 Glycoprotein 3.44 SGU

Serum, IgG, Cardiolipin antibody 8.4 GPL Serum, IgG, Cardiolipin antibody 17.45 GPL

Lupus anticoagulant by DRVVT Negative

Sr Cholesterol 211mg/dl HDL 29 mg/dl LDL 156mg/dl TGs 130 mg/dl

MRI Hips & SI joints Transient bone marrow oedema/osteopenia of bilateral hip.

PET CT Increased metabolic activity in both hip joints

Bone Scan (99mTcMDP) Increased vascularity in perfusion phase, increased accumulation in soft tissue in blood pool phase and increased uptake in bilateral Hip joints in skeletal phase scan, suggestive of CRPS Type-I.

Management

Was initially managed with Tab Etoricoxib 90mg BD, also started on Tab Sulphaslazine and Tab Methotrexate. However, when he had no symptomatic relief he was administered Inj Infliximab on 12 May 2022 and a second dose on 9 June 2022. He had excellent pain relief after the 1st dose, however after 10 days of the administration, he again began experiencing pain especially after walking. He also had pain in the knees on this occasion.

He was also administered Inj Zoledronic 4mg on 23 May 2022.

He is at present not requiring any NSAIDs over the last 1 month.

**Discussion/Results:**

The patient having presented with excruciating and debilitating pain was worked up and evaluation revealed features of bone marrow oedema on MRI which was corroborated with bone scan and PET CT imaging. The acute phase reactant CRP was also significantly elevated. The patient also gave history of early morning stiffness lasting approximately 45 mins. Hence an underlying Inflammatory process such as Spondyloarthritis(Peripheral) with enthesitis was considered. The confounding factors were the pain which worsened on mobilization, HLA B27 negative status, Rheumatoid Factor and ACCP negative status and past history of having received IV Corticosteroids for COVID infection in August 2020.

In view of the debilitating pain and a working diagnosis of Spondyloarthritis, he was started on NSAIDs along with rest, initially, followed by conventional synthetic disease modifying agents in Rheumatic disease followed by biologic synthetic disease modifying agent – Inj Infliximab.

The thought process was to avoid prolonged NSAID use to prevent the associated side effects.

However, since Avascular Necrosis of the Femoral head is a very likely possibility, the patient is planned to be kept under close follow up.

**Key learning points/Conclusion:**

Collaborative efforts between the Departments of Nuclear Medicine, Radiology, Orthopaedics and Rheumatology are crucial in the early detection and approach to cases of Bone Marrow oedema.

Avascular necrosis of head of Femur is a far more common entity and must be kept in mind even when a diagnosis of Bone Marrow oedema syndrome is being entertained.

Diagnosis of Bone Marrow oedema syndrome must be entertained only as a diagnosis of exclusion.

Continued follow up and regular imaging must be pursued rigorously in patients diagnosed with Bone Marrow oedema syndromes.

There is a requirement to document acute phase reactants such as CRP and ESR in patients diagnosed with Avascular necrosis of bone as this data could help us differentiate AVN from Primary Bone marrow oedema in the early stages.

